# Aerobic exercise inhibits oxidative stress and improves diabetic cardiomyopathy in rats by activating the PROC/PAR1/Nrf2/HO-1 signaling pathway

**DOI:** 10.3389/fphys.2026.1727186

**Published:** 2026-01-29

**Authors:** Sicong Xie, Cheng Chang, Thi Mai Tran, Zhiyi Zhou, Chenshuo Yu, Yucheng Chen, Jiayin Lin, Jiaxuan Xu, Lei Wang, Yang Zhang

**Affiliations:** 1 Department of Rehabilitation Medicine, School of Acupuncture-Moxibustion and Tuina and School of Health Preservation and Rehabilitation, Nanjing University of Chinese Medicine, Nanjing, Jiangsu, China; 2 Department of Cardiology, Kunshan Hospital of Traditional Chinese Medicine, Kunshan, Jiangsu, China

**Keywords:** aerobic exercise, bioinformatics, diabetes cardiomyopathy, oxidative stress, PROC/PAR1/Nrf2/HO-1 signaling pathway

## Abstract

Diabetic cardiomyopathy (DCM) is a serious complication of end-stage diabetes that manifests as cardiac hypertrophy and heart failure. The present study performed a bioinformatics analysis to predict possible targets for aerobic exercise to improve DCM, and animal experiments were conducted to detect the relevant mechanisms. Oxidative stress (OS)-DCM-trained differentially expressed genes (DEGs) were retrieved from the GeneCards database and a Gene Expression Omnibus microarray dataset. Subsequently, a protein-protein interaction network was constructed to screen the hub genes of the OS-DCM-trained DEGs. In addition, a model of type 2 diabetes was established using streptozotocin and a high-fat diet. Rats were divided into the control, DCM and DCM plus exercise (DCME) groups. The DCME group underwent 8 weeks of moderate-intensity treadmill training. Assessment of cardiac function, myocardial enzymes and OS-related indicators in each group. Compared with the control group, the levels of BNP, CK-MB, c-TnT, LDH, MDA, LVEF, LVIDd, and LVIDs in the DCM group were significantly increased (P < 0.05), while SOD, GSH, and LVFS were significantly decreased (P < 0.05); The above indicators were significantly improved in DCME group rats (P < 0.05). In addition, the expression levels of target genes predicted to be associated with the aerobic exercise-induced improvement of DCM were detected and western blotting was used to determine the relevant signaling pathways. Bioinformatics analysis identified nine hub genes, which, according to Kyoto Encyclopedia of Genes and Genomes enrichment analysis, were mainly involved in “IL-17 signaling pathway,” “TNF signaling pathway,” “apoptosis” and “necroptosis.” Aerobic exercise improved the heart function and myocardial enzymes of the rats in the DCM group, reduced myocardial damage, and inhibited fibrosis and OS. Detection of the nine core genes revealed that only protein C (*PROC*) met the predicted trend; *PROC* expression was lower in the DCM group than that in the control group and was higher in the DCME group than that in the DCM group (P < 0.05). Further confirmation using western blotting suggested that aerobic exercise may improve DCM by activating the PROC/proteinase-activated receptor 1 (PAR1)/nuclear factor (erythroid-derived 2)-like 2 (Nrf2)/heme oxygenase-1 (HO-1) signaling pathway. In conclusion, aerobic exercise may mitigate DCM by activating the PROC/PAR1/Nrf2/HO-1 signaling pathway. These findings could pave the way for further investigations into how exercise might regulate OS and influences DCM progression, providing novel insights into its diagnosis and prognosis.

## 1 Introduction

Type 2 diabetes has become increasingly common worldwide (Hu et al., 2013) and cardiovascular issues are the predominant causes of death among individuals with diabetes. Diabetic cardiomyopathy (DCM), a cardiac condition associated with diabetes, occurs independent of coronary artery disease and hypertension (Chavali et al., 2013). DCM is characterized by structural and functional anomalies of the heart, including myocardial hypertrophy, interstitial fibrosis, cardiomyocyte apoptosis, and impairments in diastolic and systolic functions (Huynh et al., 2014). These hallmarks of DCM contribute to the onset of heart failure and an elevated risk of mortality among affected individuals (Tribouilloy et al., 2008). Clinical studies have shown that early identification and diagnosis and timely treatment are crucial for controlling the patient’s condition (Chen et al., 2024).

DCM progression is also influenced by oxidative stress (OS) (Mohammed Yusof et al., 2018). Under physiological conditions, a number of cells continuously produce reactive oxygen species (ROS) such as superoxide radicals, hydroxyl radicals and hydrogen peroxide. ROS levels are regulated by several enzymes and physiological antioxidants including superoxide dismutase (SOD), glutathione (GSH) peroxidase, catalase and thioredoxin (Peng M. L. et al., 2022). However, excessive ROS production leads to OS, which negatively affects the functional integrity of biological tissues (Watanabe et al., 2010). Extensive experimental and clinical studies have shown that the production of ROS is increased in both types of diabetes, and that the onset of diabetes and its vascular complications, such as DCM, are associated with OS (Wu X. et al., 2022; Penckofer et al., 2002; Sano et al., 1998). Hyperglycemia, an important clinical manifestation of diabetes, is considered to produce ROS via the formation of advanced glycation end products (Mullarkey et al., 1990), altered polyol pathway activity (Williamson et al., 1993) and activation of NADPH oxidase via protein kinase C (Caturano et al., 2025).

Current studies on diabetic cardiomyopathy (DCM) have clearly established that oxidative stress is its core pathogenic mechanism: chronic hyperglycemia leads to excessive production of reactive oxygen species (ROS), which damages the myocardial antioxidant system and induces pathological abnormalities. Exercise can improve DCM by regulating oxidative stress through multiple pathways, such as reducing ROS production, upregulating the expression of superoxide dismutase (SOD), and activating the Keap1/Nrf2 pathway; among these, treadmill training not only enhances antioxidant capacity and reduces oxidative damage, but also alleviates DCM-related injury by reversing hyperacetylation of mitochondrial enzymes via the FGF21-SIRT3 axis (Liu et al., 2021). In addition, treadmill training can improve left ventricular systolic function (Libonati et al., 1985), an effect associated with the regulation of oxidative stress (OS) and enhancement of myocardial energy metabolism; this is because aerobic exercise can promote mitochondrial biogenesis, optimize calcium handling in cardiomyocytes, and reduce myocardial fibrosis, thereby providing support for the maintenance or restoration of left ventricular systolic function (Li et al., 2025; Pei et al., 2024; Zhang et al., 2024). However, studies on the targets related to the free radical scavenging system in treadmill training-mediated cardiac function protection in DCM remain scarce.

Genomics, transcriptomics, proteomics and metabolomics have generated notable amounts of data that can be analyzed by combining bioinformatics and computer science, resulting in new methods for studying the molecular mechanisms of diseases (Guo et al., 2022). Due to the rapid advancement of gene chip technology, the identification of differentially expressed genes (DEGs) and the examination of their roles have emerged as novel approaches for investigating the molecular underpinnings of disease progression (Cui et al., 2024). Notably, exercise causes systemic changes across physiology and molecular pathways hard to assess via one factor, but bioinformatics-based high-throughput technologies can detect and integrate multi-dimensional exercise-induced changes (e.g., gene expression, protein abundance, metabolites) missed by traditional single-target research, aiding in filtering core factors and clarifying key molecular drivers of exercise’s role in improving pathologies (Qi et al., 2024; Yan et al., 2017). The GSE129090 dataset was obtained in a study by Zhang et al. (2019a), which discovered that Bcl-2 was involved in the progression of cardiac hypertrophy. In addition, Grabowski et al. (2015) developed a rat model of left ventricular hypertrophy and investigated *Efcab6* as a potential candidate for left ventricular hypertrophy.

Regarding protein C (PROC), a potential effector, previous studies have shown that its activated form, activated protein C (APC), can induce the expression of protective genes in endothelial cells by activating the endothelial protein C receptor (O'Brien et al., 2006) or protease-activated receptor 1 (PAR1) in the receptor cascade (Esmon, 2006; Liu et al., 2012) further confirmed that PAR1 can activate the nuclear factor (erythroid-derived 2)-like 2 (Nrf2)/heme oxygenase-1 (HO-1) pathway, thereby exerting an anti-oxidative stress effect. These studies suggest that PROC and its related signaling pathways hold significant research value for alleviating oxidative stress in DCM.

Based on the hypothesis that “aerobic exercise improves diabetic cardiomyopathy (DCM) by regulating core genes, activating signaling pathways and inhibiting oxidative stress (OS),” this study had two core objectives: first, to identify DCM-improving core genes related to aerobic exercise from multi-omics data via bioinformatics; second, to verify aerobic exercise’s therapeutic effect on DCM via animal experiments, detect the expression of selected core genes, explore underlying mechanisms, and provide potential molecular targets with hub genes as the key core for DCM intervention.

## 2 Materials and methods

### 2.1 Data source

GSE4616, GSE6880, GSE5606 and GSE4745 gene expression profiles were obtained from the Gene Expression Omnibus (GEO) database (http://www.ncbi.nlm.nih.gov/geo), a repository of high-throughput gene expression data, hybridization arrays, chips and microarrays (Lehti et al., 2007; Van Lunteren and Moyer, 2007; Glyn-Jones et al., 2007; Gerber et al., 2006). GSE4616 is a dataset based on the GPL81 platform (Affymetrix Murine Genome U74A Version 2 Array) and includes nine mouse myocardial samples, three control samples (males), three DCM samples (males) and three DCM-trained samples (males). The DCM-trained group underwent 1 h of treadmill training daily at a speed of 21 m/min and an incline of 2.5°. Following 1 day of acclimatization training on the rodent treadmill, the mice underwent the aforementioned training regimen 5 days/week. GSE6880 is a dataset based on the GPL341 platform (Affymetrix Rat Expression 230A Array) and includes six rat myocardial samples, three control samples (males) and three DCM samples (males). GSE5606 is a dataset based on the GPL1355 platform (Affymetrix Rat Genome 230 2.0 Array) and includes 14 rat myocardial samples, seven control samples (males) and seven DCM samples (males). GSE4745 is a dataset based on the GPL85 platform (Affymetrix Rat Genome U34 Array) and includes 24 rat myocardial samples, 12 control samples (males) and 12 DCM samples (males).

The BioBase package 2.68 (https://bioconductor.org/packages/Biobase) was used to normalize the data. According to the annotation information on the platform, the probes were labeled with gene symbols, multiple probes corresponding to the same gene were randomly selected to remove duplicates and a gene expression matrix was obtained.

### 2.2 OS-related gene datasets

The GeneCards database (https://www.genecards.org/) (Xu et al., 2023) was searched using the term ‘oxidative stress’ as a screening condition to collect genes related to OS.

### 2.3 Data preprocessing and integration

The standardized expression matrix from the microarray data was obtained from the GEO datasets and depicted through a box-line plot created using the ‘ggplot2’ package (https://github.com/tidyverse/ggplot2) in R 4.2.1 (https://cran.r-project.org/bin/windows/base/old/4.2.1/). The probes were characterized using the annotation file of the dataset. Principal component analysis (PCA) was conducted to confirm the reproducibility and PCA visualizations were produced using the R package ‘ggplot2’.

The DEGs were screened using the “limma” package in R (https://bioinf.wehi.edu.au/limma/). The cutoff criteria for statistical significance were an absolute log_2_ fold change (logFC) > 1 and P < 0.05. A heat map and volcano plot of DEGs were constructed using the “ggplot” package.

### 2.4 Screening of DEGs and OS-DEGs between DCM and control samples

The “limma” package in R was used to screen for DEGs between DCM and control samples in the GSE4616, GSE6880, GSE5606 and GSE4745 datasets. P < 0.05 and |logFC|≥1 were set as the threshold values for DEG identification. Subsequently, OS-related genes were retrieved from the GeneCards database. The OS-related gene list intersected with the previously identified DEGs. The results obtained by the two methods were combined to screen OS-DEGs and their differential expression was analyzed, identifying upregulated and downregulated OS-DEGs.

### 2.5 Protein-protein interaction (PPI) network analysis

A PPI network was established using the Search Tool for the Retrieval of Interacting Genes (STRING) database (https://string-db.org), which encompassed the majority of functional interactions among the proteins encoded for by OS-DEGs. Interactions that achieved a combined score of >0.4 were considered statistically significant. The upregulated and downregulated OS-DEGs in the PPI network were examined using the STRING database.

### 2.6 Screening of DCM-trained DEGs and OS-DEGs

The “limma” package in R was used to screen for DEGs between DCM and DCM-trained samples in the GSE4616 dataset. P < 0.05 and |logFC|≥1 were set as the threshold values for DEG identification. Subsequently, lists of the aforementioned upregulated and downregulated OS-DEGs were generated, and these two lists were intersected with the DEGs between the DCM- and DCM-trained groups. The results obtained by the two methods were combined to screen OS-DCM-trained DEGs, and the differential expression of OS-DCM-trained DEGs was analyzed, identifying upregulated and downregulated OS-DCM-trained DEGs.

### 2.7 PPI network analysis of OS-DCM-trained DEGs

The PPI network was developed using the STRING database, which included nearly all functional interactions among proteins encoded by the OS-DCM-trained DEGs. Interactions with a combined score of >0.4 were regarded as statistically significant. Upregulated and downregulated OS-DCM-trained DEGs were examined using the STRING database.

### 2.8 Association analysis of OS-DCM-trained DEGs

The results of the OS-DCM-trained DEGs STRING analysis were visualized using Cytoscape (version 3.8.0) software (https://cytoscape.org/release_notes_3_8_0.html). The most closely connected modules from the PPI network (minimum required interaction score, 0.4) were selected for further analysis using the molecular complex detection plugin. The Cytoscape software plugin ‘cytoHubba’ (https://apps.cytoscape.org/apps/cytohubba) was used to filter hub genes from the entire PPI network, and to calculate the subgraph, information, local average connectivity, betweenness and closeness algorithms.

### 2.9 Gene ontology (GO) and kyoto encyclopedia of genes and genomes (KEGG) analyses

The Database for Annotation, Visualization, and Integrated Discovery (https://davidbioinformatics.nih.gov) (version 6.8) is a web-based analysis tool suite with an integrated discovery and annotation function that provides batch annotation and GO term enrichment analysis, in order to highlight the most relevant GO terms associated with related genes. The identified OS-DCM-trained DEGs were classified into three categories using GO analysis (ClusterProfiler package, https://www.bioconductor.org/packages/release/bioc/html/clusterProfiler.html): Molecular function (MF), biological process (BP) and cellular component (CC). Using Metascape (http://metascape.org), online KEGG enrichment analysis was also performed to predict the signaling pathways in which OS-DCM-trained DEGs may participate. Only terms with P < 0.05 were considered significant. The enriched pathways relevant to the current study are presented.

### 2.10 Experimental animals

Healthy 8-week-old male Sprague-Dawley rats (n = 15; weight, ∼200 ± 20 g; license SCXK 2019-0002; Hangzhou Medical College, Hangzhou, China) were housed at a constant temperature of 22 °C ± 2 °C and 40%–50% humidity, under a 12-h light/dark cycle. Rats were fed standard rodent mash for 1 week and water *ad libitum*, and were then randomly separated into two groups: Control (n = 5) and type 2 diabetes mellitus (D2M; n = 10). The control group was fed a normal diet, whereas the DCM group was fed a high-fat diet (HFD) (cat. no. D12492; Research Diets, Inc.). All animal procedures were performed in accordance with the institutional guidelines and approved by the Institutional Animal Care and Use Committee and the Ethics Committee for Science Research of the Nanjing University of Chinese Medicine (Nanjing, China; approval no. 202401A029).

### 2.11 Experimental protocol

The methodology for inducing type 2 diabetes in rats has been described previously (Peng M. L. et al., 2022). Following a 4-week period of a consistent HFD, rats in the DCM group underwent a 12-h fast. Subsequently, a 2% solution of streptozotocin (STZ; Beijing Solarbio Science & Technology Co., Ltd.), prepared in 0.01 mM citric acid buffer at pH 4.1, was administered intraperitoneally at a dosage of 30 mg/kg body weight. The control group received a single intraperitoneal injection of the same volume of 0.01 mM citric acid buffer. After a 72-h interval, 200 μL blood samples were randomly drawn from the tail vein for analysis. If blood glucose levels reached >11.1 mM, the DCM model was considered successfully established. A total of 10 rats were successfully modeled after 1 week of re-examination and removal of rats with substandard blood glucose. To measure blood glucose levels, ∼200 μL blood was collected from the tail vein of rats weekly for a total of 2 weeks, and tested using a blood glucose meter (Roche Diagnostics). The amount of blood collected was less than the recommended maximum weekly blood collection amount, which is 7.5% of the circulating blood volume. Rats with successful modeling were randomly divided into the DCM (n = 5) and DCM plus exercise (DCME; n = 5) groups.

Ten successfully modeled rats were grouped using a completely randomized design: first, the rats were numbered consecutively from 1 to 10, then 10 non-repetitive random numbers (ranging from 1 to 10) were generated by computer. After sorting the random numbers, the rats corresponding to numbers 1-5 were assigned to the DCM group (n = 5), and those corresponding to numbers 6-10 were assigned to the DCME group (n = 5). The grouping was performed by independent personnel to avoid bias.

The rats assigned to the DCME group underwent an initial week of adaptive training, which consisted of running at a speed of 10 m/min for 15 min daily. This was followed by an 8-week regimen of moderate-intensity aerobic treadmill exercise as previously described (Yang et al., 2024). The exercise parameters included a speed of 15.2 m/min and a slope of 3, corresponding to an exercise intensity of ∼58.40 ± 1.7% of VO_2_max. The sessions lasted 60 min per day and were conducted 5 days per week.

Studies have shown that 8 weeks of exercise training can achieve significant improvements in myocardial structure and function in diabetic cardiomyopathy (DCM) rats, providing sufficient time for the activation of relevant signaling pathways (Ma et al., 2025). Meanwhile, in rodent DCM models, moderate-intensity exercise has been widely confirmed to stably enhance myocardial antioxidant capacity (Wang and Feng, 2019). This intensity not only matches the clinically recommended exercise intensity for DCM patients but also avoids the risk of excessive cardiac load that may be induced by high-intensity exercise (Dias et al., 2018).

### 2.12 Insulin resistance test

Following a 12-h fasting period, serum samples were obtained from five rats each in the control group and the DCM group; briefly, 0.1 mL was collected from the tail vein of each rat, and centrifuged at 1,200 × g for 10 min at 2 °C–8 °C. The blood was allowed to clot for 30 min before serum collection. Fasting blood glucose (FBG) levels were measured using an automatic biochemical analyzer (Hitachi 7020; Hitachi, Ltd.) with a glucose oxidase kit (cat. no. ml059579; Shanghai Enzyme-linked Biotechnology Co., Ltd.). An enzyme-linked immunosorbent assay (ELISA) was used to measure fasting plasma insulin (FINS) (cat. no. HB549-Ra; Shanghai Hengyuan Biological Technology Co., Ltd.) using a microplate reader (WellScan MK3; LabSystems Diagnostics Oy). The insulin sensitivity index (ISI) and homeostatic model assessment of insulin resistance (HOMA-IR) were calculated using the following formulae: ISI = −log(FPG × FINS); HOMA-IR = (FPG × FINS)/22.5.

### 2.13 Echocardiogram

After 8 weeks of exercise, echocardiography was performed using a VINNO6 high-resolution imaging system [VINNO Technology (Suzhou) Co., Ltd.] following isoflurane anesthesia. The anesthesia protocol included induction with 4% isoflurane in 100% oxygen (1 L/min flow rate) using a precision evaporator and nasal cone, with anesthesia achieved within 2 min. Isoflurane concentration was maintained at 2% to ensure stable hemodynamics and to reduce cardiorespiratory depression. All procedures followed the institutional animal care guidelines and heating pads were used to maintain body temperature at 37 °C. Left ventricular ejection fraction (LVEF), left ventricular fractional shortening (LVFS), left ventricular internal diastolic dimension (LVIDd) and left ventricular internal dimension systole (LVIDs) were measured (Li et al., 2021).

### 2.14 Sample collection

After 8 weeks of exercise, all rats were euthanized. For euthanasia of Sprague-Dawley rats, sodium pentobarbital was administered intraperitoneally at a dose of 150 mg/kg. After sodium pentobarbital injection, death was confirmed by checking that rats were immobile, lacked a pain response when subjected to a toe pinch, were confirmed to lack a heartbeat and breathing, and exhibited dilated pupils. This protocol ensured rapid and humane euthanasia while complying with American Veterinary Medical Association guidelines (Gu et al., 2016).

In addition, for blood collection, pentobarbital sodium (50 mg/kg rat body weight; 1% solution concentration) was administered intraperitoneally to all rats in each group and the disappearance of corneal reflexes and stabilization of thoracic respiration were observed (Clifford et al., 2018). Subsequently, ∼10 mL blood was obtained from the abdominal aorta from rats weighing 250 g, and the rats were immediately euthanized upon completion of this procedure, by sodium pentobarbital overdose. These blood samples were then centrifuged at 1,200 × g for 15 min at 25 °C before the supernatant was collected. Upon collection of blood samples, the hearts of the rats were excised and rinsed with cold 0.9% saline. The surrounding vessels and connective tissues, along with the atrial and right ventricular tissues, were carefully removed. The procured cardiac tissue was then divided into two portions: Apical sections were swiftly frozen in liquid nitrogen (−196 °C) for subsequent western blot analysis and the remaining tissue was preserved in 10% neutral-buffered formalin for fixation at room temperature for 4 h.

### 2.15 Histological evaluation

The excised heart tissues were embedded in paraffin and the tissue samples were sliced into 5-μm sections for later staining with hematoxylin and eosin (H&E) at 90 °C for 30 min. Masson’s trichrome staining was also performed on the 5-μm sections to assess interstitial fibrosis (Zhang et al., 2019b). Images were obtained using a light microscope (BX51; Olympus Corporation) and a digital imaging system (DP71; Olympus Corporation). H&E- and Masson’s trichrome-stained tissue sections were examined at ×200 magnification.

### 2.16 Assessment of biochemical parameters

Serum levels of brain natriuretic peptide (BNP; cat. no. SEKR-0058; Beijing Solarbio Science & Technology Co., Ltd.), creatine kinase MB (CK-MB; cat. no. SEKR-0059; Beijing Solarbio Science & Technology Co., Ltd.), cardiac troponin T (c-TnT; cat. no. SEKR-0047; Beijing Solarbio Science & Technology Co., Ltd.), malondialdehyde (MDA; cat. no. SP30131; Wuhan Saipei Biotechnology Co., Ltd.), lactate dehydrogenase (LDH; cat. no. ml106660; Shanghai Enzyme-linked Biotechnology Co., Ltd.), GSH (cat. no. SP12673; Wuhan Saipei Biotechnology Co., Ltd.) and SOD (cat, no. SP12914; Wuhan Saipei Biotechnology Co., Ltd) were measured using ELISA kits, according to the manufacturer’s instructions.

### 2.17 Reverse transcription-quantitative polymerase chain reaction (RT-qPCR)

Total RNA was extracted from the myocardium using an RNA extraction kit (cat. no. R1051; Guangzhou Dongsheng Biotech Co., Ltd.), and was used to synthesize cDNA using All-in-One first-strand cDNA Synthesis SuperMix for qPCR (TransGen Biotech Co. Ltd.) according to the manufacturer’s protocol. A Tip Green qPCR SuperMix kit (TransGen Biotech Co., Ltd.) was used for qPCR according to the manufacturer’s instructions, with GAPDH used as the housekeeping gene. The thermocycling conditions were as follows: Pre-denaturation at 95 °C for 3 min, followed by 40 cycles of denaturation at 95 °C for 10 s followed by annealing/extension at 60 °C for 30 s. The mRNA expression levels of the target genes (Table 1) were calculated using the 2^−ΔΔCq^ method.

**TABLE 1:** Primer sequences for reverse transcription-quantitative PCR.

Gene	Sequence, 5′-3′	References sequence
*Rattus FGG*		NM_012559.2
Forward	CCGCCTGACCTATGCCTACTTC	
Reverse	CATCCACCAGCCAGATCCATCC	
*Rattus HGD*		NM_001012145.1
Forward	CCATTGCTGACTTTGTGATCTTCCC	
Reverse	GAATCCACCTTGCTTTGCCTCATAG	
*Rattus PROC*		NM_012803.1
Forward	GAGCAACAGCGACAACGACATC	
Reverse	GTTCCTTCTGCCATCCTTGACTTTG	
*Rattus TRADD*		NM_001100480.1
Forward	ACTGGAGTTGCGTGCTGGTG	
Reverse	GTCGGGCTTCTGGGCTAAGATG	
*Rattus GC*		NM_012564.3
Forward	TATGTGGAGCCGACGAATGATGAG	
Reverse	AAGTACAGCAGGAGCCAACCATAG	
*Rattus CCNA1*		NM_001011949.1
Forward	TGACCGTTCCAACCACCAACC	
Reverse	TGCTGCTACCAAGGAAGGAAGATAC	
*Rattus SPAG5*		NM_001044224.1
Forward	GGAAGTAGGCACCAAGGACAGTAC	
Reverse	GCAAGACAAGGGCGTTCAACAG	
*Rattus CASP8*		NM_022277.2
Forward	CGGTGCCTGTGCCTGATGAG	
Reverse	GCGTGGGATCTCGGTAGGAAAC	
*Rattus TOP2A*		NM_001393768.1
Forward	ACAGTCGCAAGAGGAAGCCATC	
Reverse	GAGGAGCCACAGTGTCATCTAAGTC	
*Rattus GAPDH*		NM_017008.4
Forward	AGTGCCAGCCTCGTCTCATA	
Reverse	ATCCGTTCACACCGACCTTC	

*FGG*, fibrinogen γ chain; *HGD*, homogentisate 1,2-dioxygenase; *PROC*, protein C; *TRADD*, tumor necrosis factor receptor type 1-associated DEATH domain protein; *GC*, vitamin D-binding protein; *CCNA1*, cyclin A1; *SPAG5*, sperm-associated antigen 5; *CASP8*, caspase-8; *TOP2A*, DNA topoisomerase 2α.

### 2.18 Concentrations of activated protein C (APC)

The concentration of APC in the tissue homogenate samples was determined using a commercially available Rat APC ELISA Kit (cat. no. XYR121; XYbio) according to the manufacturer’s instructions. Before the test, the frozen serum samples were thawed and centrifuged again at 1,000 × g for 15 min at 4 °C and all reagents in the ELISA kit were brought to room temperature. The serum supernatant was added and the test performed according to the manufacturer’s instructions.

### 2.19 Western blot analysis

Western blot analysis was used to determine the protein levels in rat myocardial tissue samples. Briefly, mouse myocardial tissue samples were rapidly homogenized in 200 μL RIPA lysis buffer (cat. no. P0013C; Beyotime Institute of Biotechnology) and protein levels were determined using the BCA method. Subsequently, 20 μg protein/lane were separated by sodium dodecyl sulfate-polyacrylamide gel electrophoresis on a 10% gel, and the proteins were then transferred onto nitrocellulose membranes. The membranes were blocked with 5% non-fat dry milk in Tris-buffered saline for 60 min at room temperature and then incubated with anti-PROC (1:1,000; cat. no. MA5-35528; Thermo Fisher Scientific, Inc.), anti-tumor necrosis factor receptor type 1-associated DEATH domain protein (TRADD) (1:500) (cat. no. 703356; Thermo Fisher Scientific, Inc.), anti-caspase-8 (CASP8) (1:1,000; cat. no. MA1-41280; Thermo Fisher Scientific, Inc.), anti-cleaved CASP8 (1:1,000; cat. no. PA5-99435; Thermo Fisher Scientific, Inc.), anti-proteinase-activated receptor 1 (PAR1) (1:1,000; cat. no. PA5-116040; Thermo Fisher Scientific, Inc.), anti-nuclear factor (erythroid-derived 2)-like 2 (Nrf2; 1:1,000; cat. no. PA5-27882; Thermo Fisher Scientific, Inc.), anti-heme oxygenase-1 (HO-1; 1:1,000; cat. no. PA5-77833; Thermo Fisher Scientific, Inc.) and anti-GAPDH (1:1,000; cat. no. MA5-35235; Thermo Fisher Scientific, Inc.) antibodies. The membranes were incubated overnight at 4 °C and washed three times before being incubated with the appropriate HRP-conjugated anti-rabbit (1:5,000; cat. no. 32460; Thermo Fisher Scientific, Inc.) or anti-mouse secondary antibodies (1:5,000; cat. no. 31431; Thermo Fisher Scientific, Inc.) for 60 min at room temperature. Enhanced chemiluminescent reagents (cat. no. 34098CN; Thermo Fisher Scientific, Inc.) were used to visualize protein bands. A digital gel imaging system was used to examine the protein expression levels (Alpha Imager2200 3.2; ProteinSimple).

### 2.20 Statistical analysis

All data are expressed as mean ± SD and were analyzed using SPSS 21.0 (IBM, United States). Normality and homoscedasticity were assessed using the Shapiro-Wilk test and Levene’s test, respectively. For data meeting the assumptions of normality and equal variance, an unpaired Student’s t-test was employed for two-group comparisons, and one-way ANOVA followed by Tukey’s post-hoc test was used for multiple group comparisons. A value of P < 0.05 was considered statistically significant.

## 3 Results

### 3.1 Screening of OS-DEGs between DCM and control groups

**FIGURE 1:**
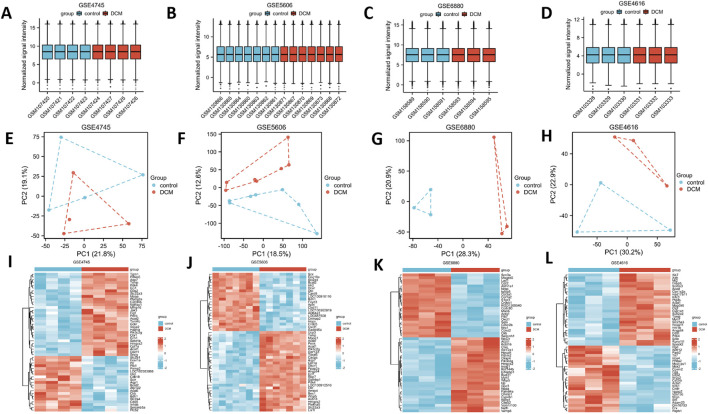
Differentially expressed genes in control and DCM samples. Normalized expression matrices for **(A)** GSE4745, **(B)** GSE5606, **(C)** GSE6880 and **(D)** GSE4616. Principal component analysis diagrams of the **(E)** GSE4745, **(F)** GSE5606, **(G)** GSE6880 and **(H)** GSE4616 datasets. Part of the hierarchical clustering heat maps of the **(I)** GSE4745, **(J)** GSE5606, **(K)** GSE6880 and **(L)** GSE4616 datasets showing the differentially expressed genes in the control and DCM samples. DCM, diabetic cardiomyopathy.

Four datasets were chosen to examine variations in gene expression between the DCM and control groups. The expression matrices for the GSE4616, GSE6880, GSE5606 and GSE4745 datasets were normalized, resulting in box plots that displayed the distribution trends as straight lines (Figures 1A–D). PCA was conducted on the four datasets to evaluate the consistency of the data within each group. The results indicated a high level of repeatability (Figures 1E–H). Figures 1I–L shows the partial gene heat maps of the four datasets.

**FIGURE 2:**
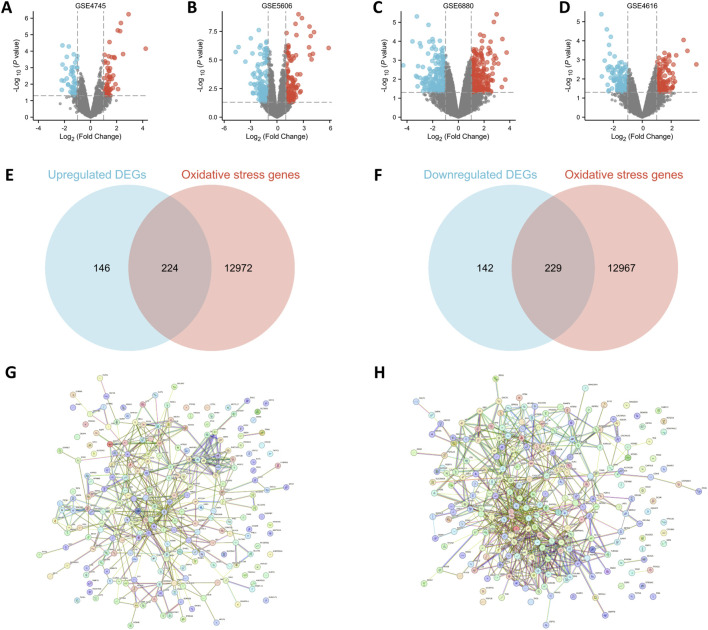
DEGs and OS-related genes of the GSE4745, GSE5606, GSE6880 and GSE4616 datasets. **(A–D)** Volcano plots of DEGs. The red nodes represent upregulated DCM-related DEGs with P < 0.05 and logFC>1; the blue nodes represent downregulated DCM-related DEGs with P < 0.05 and logFC<-1. **(E,F)** Venn diagrams of 224 upregulated OS-DEGs and 229 downregulated OS-DEG. **(G,H)** Protein-protein interaction network diagrams of OS-DEGs. Not sig, not significant; DEGs, differentially-expressed genes; OS, oxidative stress.

After screening with the threshold of an adjusted |logFC| > 1 and P < 0.05, 105 DEGs (58 upregulated and 47 downregulated in the DCM group) were identified in the GSE4745 dataset (Supplementary Table S1), 90 DEGs (45 upregulated and 45 downregulated in the DCM group) in the GSE5606 dataset (Supplementary Table S2), 382 DEGs (198 upregulated and 184 downregulated in the DCM group) in the GSE6880 dataset (Supplementary Table S3) and 238 DEGs (111 upregulated and 127 downregulated in the DCM group) in the GSE4616 dataset (Supplementary Table S4). Volcano plots of the DEGs in the four datasets are shown in Figures 2A–D. A total of 370 upregulated and 371 downregulated genes were identified by merging the upregulated and downregulated genes from the four datasets. The GeneCards database was used to cross-reference genes encoding OS proteins with the DEGs. A total of 13,196 related genes were identified in the GeneCards database (Supplementary Table S5), and 224 upregulated and 229 downregulated OS-DEGs were identified (Figures 2E,F). A PPI network was created by determining the interactions between upregulated and downregulated OS-DEGs. The PPI network showed that most OS-DEGs interacted with each other, and that the closer they were to the center of the network, the more genes they interacted with Figures 2G,H.

### 3.2 Screening of OS-DCM-trained DEGs

**FIGURE 3:**
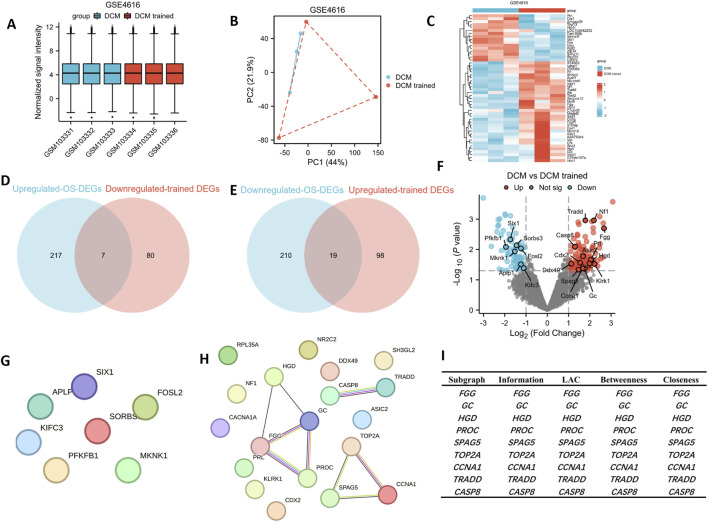
DEGs in DCM and DCM-trained samples. **(A)** Normalized expression matrices of the GSE4616 dataset. **(B)** Principal component analysis diagrams of the GSE4616 dataset. **(C)** Part of the hierarchical clustering heat map of the GSE4616 dataset DEGs in DCM and DCM-trained samples. Venn diagrams of **(D)** seven upregulated OS-DCM-trained DEGs and **(E)** 19 downregulated OS-DCM-trained DEGs. **(F)** Volcano plot of seven upregulated OS-DCM-trained DEGs and 19 downregulated OS-DCM-trained DEGs. **(G,H)** Protein-protein interaction network diagrams of OS-DCM-trained DEGs. **(I)** Nine hub genes of upregulated OS-DCM-trained DEGs using Cytoscape. DCM, diabetic cardiomyopathy; DEGs, differentially-expressed genes; *FGG*, fibrinogen γ chain; *HGD*, homogentisate 1,2-dioxygenase; *PROC*, protein C; *TRADD*, tumor necrosis factor receptor type 1-associated DEATH domain protein; *GC*, vitamin D-binding protein; *CCNA1*, cyclin A1; *SPAG5*, sperm-associated antigen 5; *CASP8*, caspase-8; *TOP2A*, DNA topoisomerase 2α; Not sig, not significant; LAC, local average connectivity.

The GSE4616 dataset was the only one assessed in the present study that used aerobic exercise intervention on DCM. The expression matrices within the GSE4616 dataset were normalized, resulting in box plots that exhibited linear distribution trends (Figure 3A). To evaluate intragroup data reproducibility, PCA was conducted on the dataset, which revealed a high degree of repeatability (Figure 3B). Figure 3C shows a partial heat map of the dataset.

After screening using the thresholds of adjusted |logFC| > 1 and P < 0.05, 204 DCM-trained DEGs (117 upregulated and 87 downregulated) were identified in the GSE4616 dataset (Supplementary Table S6). Subsequently, using a Venn diagram, the upregulated OS-DEGs were intersected with the downregulated DCM-trained DEGs, resulting in seven DEGs (Figure 3D). Simultaneously, 19 DEGs were identified by intersecting the downregulated OS-DEGs with the upregulated DCM-trained DEGs (Figure 3E). A volcano plot illustrating the DEGs identified by OS-DCM training in the aforementioned datasets is shown in Figure 3F. The PPI network was created by determining the interactions between the downregulated (Figure 3G) and upregulated OS-DCM-trained DEGs (Figure 3H).

### 3.3 Identification of hub genes

The PPI results showed no association between the downregulated OS-DCM-trained DEGs (Figure 3G). Therefore, further research did not focus on these genes and instead investigated the upregulated OS-DCM-trained DEGs. The STRING analysis data were imported into Cytoscape and genes with scores were designated as hub genes. The top hub genes were identified using four different algorithms. An upshot diagram of the results of these algorithms revealed nine common hub genes: Fibrinogen γ chain (*FGG*), homogentisate 1,2-dioxygenase (*HGD*), *PROC*, *TRADD*, vitamin D-binding protein (*GC*), cyclin A1 (*CCNA1*), sperm-associated antigen 5 (*SPAG5*), CASP8 and DNA topoisomerase 2α (*TOP2A*) (Figure 3I).

### 3.4 GO and KEGG analyses

**FIGURE 4:**
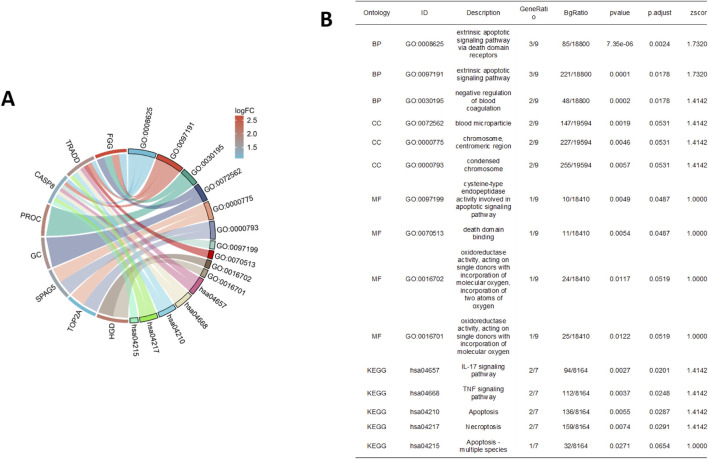
GO and KEGG analyses. **(A,B)** GO and KEGG pathway enrichment analyses. GO, Gene Ontology; KEGG, Kyoto Encyclopedia of Genes and Genomes; logFC, log_2_ fold change; BP, biological process; MF, molecular function; CC, cellular component; *FGG*, fibrinogen γ chain; *HGD*, homogentisate 1,2-dioxygenase; *PROC*, protein C; *TRADD*, tumor necrosis factor receptor type 1-associated DEATH domain protein; *GC*, vitamin D-binding protein; *SPAG5*, sperm-associated antigen 5; *CASP8*, caspase-8; *TOP2A*, DNA topoisomerase 2α.

GO and KEGG enrichment analyses of the upregulated OS-DCM-trained DEG hub genes were used to investigate their functions (Figures 4A,B). The ClusterProfiler package (https://www.bioconductor.org/packages/release/bioc/html/clusterProfiler.html) enriched GO function was used to determine the BP, CC and MF terms the hub genes were enriched in. The most enriched GO categories included “extrinsic apoptotic signaling pathway via death domain receptors,” “extrinsic apoptotic signaling pathway,” “cysteine-type endopeptidase activity involved in apoptotic signaling pathway,” “oxidoreductase activity, acting on single donors with incorporation of molecular oxygen” and “oxidoreductase activity, acting on single donors with incorporation of molecular oxygen, incorporation of two atoms of oxygen.” Hub genes were mainly involved in apoptotic processes, such as “IL-17 signaling pathway,” “TNF signaling pathway,” “apoptosis” and “necroptosis” in the KEGG enrichment analysis.

### 3.5 Effects of aerobic exercise on body weight and blood glucose in diabetic rats

**FIGURE 5:**
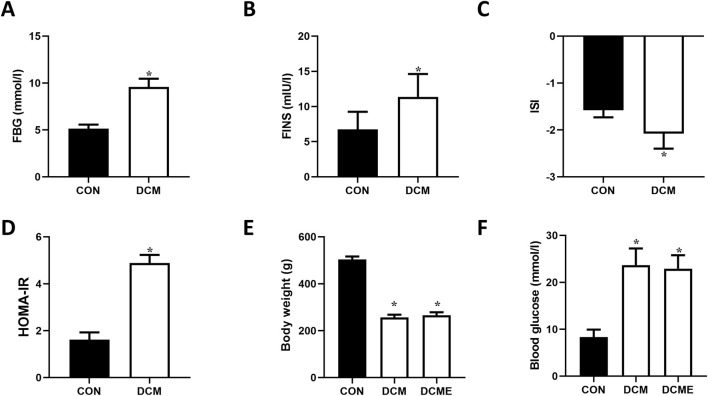
Changes in insulin sensitivity, body weight and blood glucose of rats with DCM. **(A)** FBG levels. **(B)** FINS levels. **(C)** ISI. **(D)** HOMA-IR. **(E)** Changes in rat body weight. **(F)** Changes in FBG. Data are presented as the mean ± SD (n = 5). ^*^P < 0.05 vs. CON group. DCM, diabetic cardiomyopathy; FBG, fasting blood glucose; FINS, fasting plasma insulin; ISI, insulin sensitivity index; HOMA-IR, homeostatic model assessment of insulin resistance; CON, control; DCME, DCM plus exercise.

As shown in Figures 5A–D, rats in the DCM group exhibited significantly elevated FBG and FINS levels compared with those in the control group (both P < 0.05), along with significantly reduced ISI (P < 0.05) and increased HOMA-IR (P < 0.05). These findings suggested heightened insulin resistance in the DCM group, demonstrating that the current animal model aligned with the characteristics of type 2 diabetes.

Changes in the body weight of the rats are shown in Figure 5E. Compared with in the control group, the weights of rats in the DCM and DCME groups were significantly reduced (P < 0.05). Rats in the DCME group were heavier than those in the DCM group; however, the difference was not statistically significant. Figure 5F shows changes in blood glucose levels. Compared with those in the DCM group, blood glucose levels were not significantly different in the DCME group (P > 0.05).

### 3.6 Effects of aerobic exercise on serum myocardial enzymes levels

**FIGURE 6:**
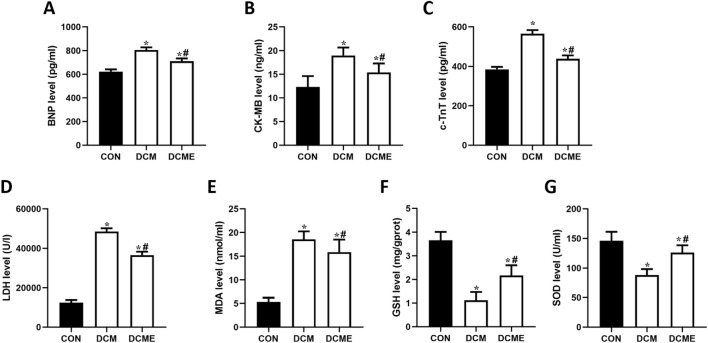
Effects of aerobic exercise on myocardial enzymes and oxidative stress indexes in rats with DCM. Representative **(A)** BNP, **(B)** CK-MB, **(C)** c-TnT and **(D)** LDH levels. **(E)** MDA content. **(F)** GSH and **(G)** SOD levels. Data are presented as the mean ± SD (n = 5). ^*^P < 0.05 vs. CON group; ^#^P < 0.05 vs. DCM group. BNP, brain natriuretic peptide; CK-MB, creatine kinase MB; c-TnT, cardiac troponin T; LDH, lactate dehydrogenase; MDA, malondialdehyde; GSH, glutathione; SOD, superoxide dismutase; CON, control; DCM, diabetic cardiomyopathy; DCME, DCM plus exercise.

As shown in Figures 6A–D, the serum levels of LDH, CK-MB, c-TnT and BNP were significantly elevated in the DCM group compared with those in the control group (P < 0.05). However, exercise training in the DCME group led to a significant reduction in LDH, CK-MB, c-TnT and BNP levels compared with those in the DCM group (P < 0.05).

### 3.7 Effects of aerobic exercise on myocardial OS in diabetic rats

Compared with that in the control group, the MDA content in the DCM group was significantly increased (P < 0.05), and the MDA content in the DCME group was significantly lower than that in the DCM group (P < 0.05), but was still significantly higher than that in the control group (P < 0.05) (Figure 6E). Furthermore, compared with those in the control group, the activities of SOD and GSH were significantly reduced in the DCM group (P < 0.05); however, compared with those in the DCM group, the SOD and GSH activities were significantly higher in the DCME group (P < 0.05) but remained significantly lower than those in the control group (P < 0.05) (Figures 6F,G).

### 3.8 Effects of aerobic exercise on myocardial pathological abnormalities

**FIGURE 7:**
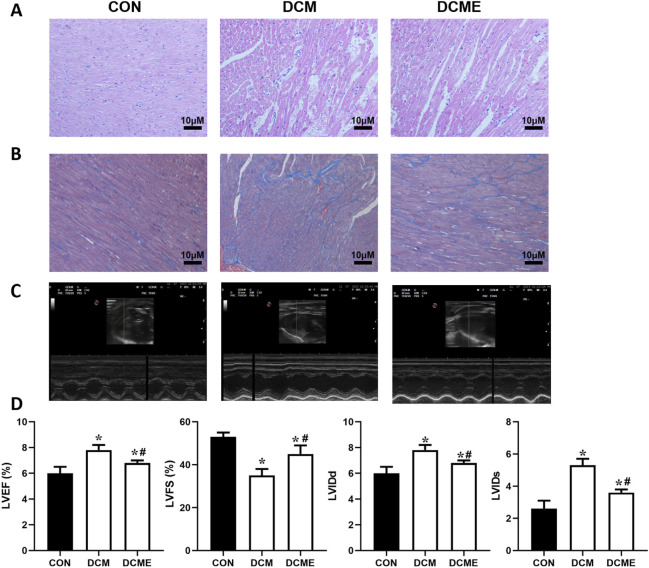
Effects of exercise training on histopathological and echocardiographic abnormalities in myocardial tissue. **(A)** H&E staining (×200 magnification) showed irregular arrangement of myocardial cells, uneven distribution of cytoplasm and ruptured myocardial fibers in the DCM group, whereas exercise training attenuated these histopathological changes. **(B)** Masson’s trichrome staining (×200 magnification) showed irregular and noticeably increased interstitial collagen fibers (blue region) in the DCM group. **(C)** Echocardiography of rats in each group. **(D)** LVEF, LVFS, LVIDd and LVIDs in each group of rats. Data are presented as the mean ± SD (n = 3). ^*^P < 0.05 vs. CON group; ^#^P < 0.05 vs. DCM group. H&E, hematoxylin and eosin; CON, control; DCM, diabetic cardiomyopathy; DCME, DCM plus exercise; LVEF, left ventricular ejection fraction; LVFS, left ventricular fractional shortening; LVIDd, left ventricular diastolic dimension; LVIDs, left ventricular systolic dimension.

After H&E staining (Figure 7A), myocardial cells in the control group appeared to be compact and arranged in an orderly manner, with bright red cytoplasm and centrally located oval nuclei. No dissolved muscle fibers, vacuolar degeneration or mononuclear cell infiltration were observed. However, in the DCM group, myocardial cells were disordered, and uneven cytoplasmic distribution, ruptured myocardial fibers and irregular nuclei were observed. The DCME group demonstrated reduced myocardial injury following moderate-intensity exercise training. Masson’s trichrome staining (Figure 7B) revealed well-organized collagen fibers and no notable myocardial interstitial collagen deposition in the control group. By contrast, the DCM group displayed disorganized myocardial cells and a marked increase in interstitial collagen fibers within both intercellular and perivascular spaces. The DCME group showed reduced collagen fiber content compared with in the DCM group.

### 3.9 Effect of aerobic exercise on echocardiography

Compared with those in the control group, the LVEF and LVFS of the DCM group were significantly decreased, but the LVIDd and LVIDs were significantly increased, indicating impaired cardiac contractile function in the DCM group (Figures 7C,D). In addition, compared with those in the DCM group, the DCME group had significantly increased LVEF and LVFS, and decreased LVIDd and LVIDs, thus indicating improved cardiac contractile function (Cheng et al., 2022; Wu et al., 2024).

### 3.10 RT-qPCR detection of the expression of hub genes in heart tissues

**FIGURE 8:**
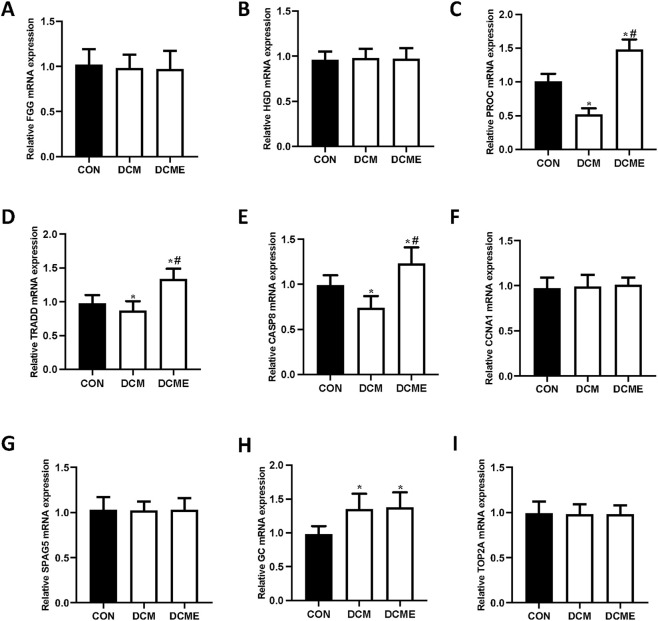
Effect of aerobic exercise on the expression levels of predicted genes in rats with DCM. Representative **(A)** *FGG*, **(B)** *HGD*, **(C)** *PROC*, **(D)** *TRADD*, **(E)** *CASP8*, **(F)** *CCNA1*, **(G)** *SPAG5*, **(H)** *GC* and **(I)** *TOP2A* mRNA expression levels. Data are presented as the mean ± SD (n = 3). ^*^P < 0.05 vs. CON group; ^#^P < 0.05 vs. DCM group. CON, control; DCM, diabetic cardiomyopathy; DCME, DCM plus exercise; *FGG*, fibrinogen γ chain; *HGD*, homogentisate 1,2-dioxygenase; *PROC*, protein C; *TRADD*, tumor necrosis factor receptor type 1-associated DEATH domain protein; *GC*, vitamin D-binding protein; *CCNA1*, cyclin A1; *SPAG5*, sperm-associated antigen 5; *CASP8*, caspase-8; *TOP2A*, DNA topoisomerase 2α.

RT-qPCR was used to examine the expression levels *of FGG, HGD, PROC, TRADD, GC, CCNA1, SPAG5, CASP8* and *TOP2A* in different samples (Figures 8A–I). According to bioinformatics predictions, compared with those in the control group, the target genes in the DCM group would be significantly reduced. By contrast, compared with in the DCM group, the target genes in the DCME group rats would be significantly elevated. Only *PROC, TRADD* and *CASP8* conformed to the predicted patterns.

### 3.11 Western blot detection of the expression of hub genes in heart tissues

**FIGURE 9:**
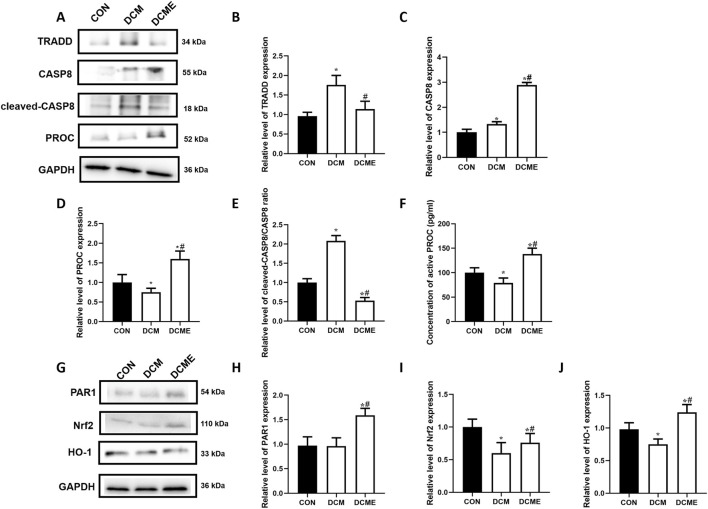
Effect of aerobic exercise on the expression levels of predicted proteins and of proteins in the PROC/PAR1/Nrf2/HO-1 signaling pathway in rats with DCM. **(A)** Representative TRADD, cleaved-CASP8, CASP8 and PROC levels; GAPDH was used as an internal control. **(B–E)** Representative TRADD and PROC ratios to GAPDH protein, and cleaved-CASP8/CASP8 ratio. **(F)** Representative active PROC levels. **(G)** Representative PAR1, Nrf2 and HO-1 levels; GAPDH was used as an internal control. **(H–J)** Representative PAR1, Nrf2 and HO-1 to GAPDH protein ratios. Data are presented as the mean ± SD (n = 3). ^*^P < 0.05 vs. CON group; ^#^P < 0.05 vs. DCM group. PROC, protein C; PAR1, proteinase-activated receptor 1; Nrf2, nuclear factor (erythroid-derived 2)-like 2; HO-1, heme oxygenase-1; TRADD, tumor necrosis factor receptor type 1-associated DEATH domain protein; CASP8, caspase-8; CON, control, DCM, diabetic cardiomyopathy; DCME, DCM plus exercise.

Compared with that in the control group, the DCM group showed a significant decrease in PROC expression in cardiac tissues (Figures 9A,D). By contrast, compared with in the DCM group, the DCME group showed a significant increase in PROC. Compared with in the control group, TRADD expression was significantly elevated in cardiac tissue from the DCM group (Figures 9A,B). Conversely, TRADD expression was markedly reduced in the DCME group relative to the DCM group. Compared with in the control group, CASP8 expression was significantly increased in cardiac tissue from the DCM group; CASP8 expression was further increased in the DCME group relative to the DCM group (Figures 9A,C). Compared with in the control group, the cleaved-CASP8/CASP8 ratio was significantly increased in cardiac tissue from the DCM group, whereas it was decreased in the DCME group relative to the DCM group (Figures 9A,E).

### 3.12 Concentrations of serum APC

As shown in Figure 9F, compared with that in the control group, the APC content in the DCM group was significantly lower than that in the control group (P < 0.05), whereas the APC content in the DCME group was significantly higher than that in the DCM group (P < 0.05).

### 3.13 Western blot detection of the expression of PROC/PAR1/Nrf2/HO-1 signaling pathway proteins in heart tissues

As shown in Figures 9G–J, compared with those in the control group, the protein levels of Nrf2 and HO-1 were significantly reduced in the DCM group (P < 0.05), whereas the levels of PAR1, Nrf2 and HO-1 were significantly increased in the DCME group compared with those in the DCM group (P < 0.05).

## 4 Discussion

In this study, differentially expressed genes related to OS and DCM were screened from 4 GEO datasets. A PPI network was constructed, leading to the identification of 9 hub genes (e.g., PROC). Animal experiments confirmed that 8 weeks of aerobic exercise improved cardiac function, alleviated injury and OS in DCM rats, and only the expression of PROC was consistent with the prediction. Finally, it was verified that aerobic exercise exerts its effect by activating the PROC/PAR1/Nrf2/HO-1 pathway. This study is the first to identify PROC as a key node in aerobic exercise-regulated DCM, filling the gap in genetic targets and providing a new direction for DCM intervention.

DCM is the leading cause of mortality in patients with diabetes, and no specific treatment is currently available in clinical practice (Livak and Schmittgen, 2001; Palomer et al., 2018). Regular exercise improves blood glucose control, lowers cardiovascular risk factors, aids in weight loss and improves overall wellbeing (Gilbert and Krum, 2015). The present study investigated the modulatory role and antioxidant potential of aerobic exercise in diabetes-induced myocardial injury.

Bioinformatics analysis was used to identify potential DEGs (She et al., 2025). The top hub genes were identified using four different algorithms. An upshot diagram of the results of these algorithms revealed nine common hub genes: *FGG, HGD, PROC, TRADD, GC, CCNA1, SPAG5, CASP8* and *TOP2A*. GO and KEGG enrichment analyses were used to investigate the functions of the upregulated OS-DCM-trained DEG hub genes. The most enriched GO categories included “extrinsic apoptotic signaling pathway via death domain receptors,” “extrinsic apoptotic signaling pathway” and “cysteine-type endopeptidase activity involved in apoptotic signaling pathway.” Hub genes were mainly involved in apoptotic processes, such as “IL-17 signaling pathway,” “TNF signaling pathway,” “apoptosis” and “necroptosis” in the KEGG enrichment analysis. Some genes involved in DCM that are differentially expressed in response to OS have been studied previously. American Diabetes Association (2012) demonstrated that the activated nuclear catenin/c-Myc axis is responsible for oxidative cardiac impairment. In addition, Liu et al. (2017) discovered that excessive ROS production in DCM can activate the TLR-4/MyD-88/CASP8/CASP3 signaling pathway, leading to cardiomyocyte apoptosis. Subsequently, it was confirmed that aerobic exercise improved the expression of central DCM genes in rats.

In the present study, 8 weeks of treadmill exercise reduced blood glucose levels in diabetic rats; however, the improvement observed was not statistically significant compared with the DCM group. This suggested that 8 weeks of aerobic exercise can partially mitigate diabetes-induced hyperglycemia. In patients with type 2 diabetes, increased blood glucose is mainly associated with reduced sensitivity to insulin (Liu et al., 2015; Su et al., 2023). In patients with diabetes, insufficient insulin sensitivity leads to persistently elevated blood glucose levels. Aerobic exercise enhances insulin sensitivity and facilitates the translocation of glucose transporter 4 to the cell membrane, promoting glucose uptake into cells, thereby lowering blood glucose levels (Galicia-Garcia et al., 2020). Way et al. (2016) also showed that the weight of diabetic rats in a D2M group was markedly decreased, along with a notable increase in FBG levels.

Cardiac function and myocardial enzyme levels were assessed in each group. Compared with those in the control group, the DCM group exhibited significantly elevated cardiac function and myocardial enzyme levels, indicating heart damage. These indicators significantly improved in the DCME group. Similarly, both H&E and Masson’s trichrome staining showed that aerobic exercise improved the myocardium of the DCM rats. Wang et al. (2019) a used a HFD and STZ to induce DCM, and disrupted myocardial alignment and interstitial fibrosis were observed. In addition, Wu S. et al. (2022) demonstrated the presence of myocardial fibrosis in a rat model of DCM using Masson’s trichrome staining and Sirius red staining techniques. These previous findings indicated that STZ and HFD effectively replicate DCM in a rat model, and that aerobic exercise may improve cardiac function and enzyme levels in rats with DCM.

OS is associated with the occurrence and progression of pathological structural and functional changes in DCM (Ren et al., 2020; Faria and Persaud, 2017). Under DCM conditions, antioxidant factors such as SOD and GSH are markedly reduced in the heart tissue, whereas reactive MDA generation, which is responsible for cellular OS, is notably increased (Liao et al., 2017; Wilson et al., 2018). Thus, therapeutic molecules and methods that target intracellular OS represent potential DCM treatment strategies. The present study found that aerobic exercise reduced MDA levels in rat myocardial tissues, increased SOD and GSH levels, and thus conferred antioxidant stress resistance. Kowluru and Mishra (2015) reported that aerobic and resistance exercises may improve functional capacity and maximum load-carrying capacity, respectively. Furthermore, Gomes et al. (2020) reported that aerobic exercise can mitigate OS and cell death in mitochondria through the modulation of the Nrf2/glycogen synthase kinase 3β signaling pathway, thus improving cognitive impairment observed in an aging model induced by D-galactose.

The predicted hub genes of the present study were tested and only the mRNA and protein expression levels of PROC were revealed to match the trend predicted by bioinformatics analysis. Aerobic exercise may exert a cardioprotective effect by regulating PROC to improve DCM. APC, a serine protease belonging to the trypsin family, is produced during the initial phase of blood coagulation through limited proteolysis of PROC by thrombin, which binds to endothelial thrombomodulin (Xie et al., 2024; Esmon and Owen, 1984). APC is a multifunctional enzyme that serves an important role in the regulation of blood coagulation, inflammation and apoptosis. It exhibits anti-inflammatory and cytoprotective properties that contribute to its diverse physiological effects (Leon et al., 2022; Biswas et al., 2024). In the present study, compared with in the control group, the APC content in the DCM group was significantly decreased, whereas the APC content in the DCME group was significantly higher than that in the DCM group, indicating that aerobic exercise significantly increased the levels of APC, thus serving a role in alleviating DCM.

Previous studies have shown that APC induces the expression of protective genes in endothelial cells by activating endothelial protein C receptor (O'Brien et al., 2006) or the receptor cascade PAR1 (Esmon, 2006). Previous studies have shown that the PAR1 and PAR3 subtype receptors serve a role in neuroprotection, particularly in response to NMDA- and staurosporin-induced neuronal apoptosis (Guo et al., 2004). Liu et al. (2012) demonstrated that PAR1 can activate the Nrf2/HO-1 signaling pathway, thereby exerting antioxidant stress resistance. In addition, Peng H. et al. (2022) found that GF1 may reduce triptolide cytotoxicity in HL-7702 cells by activating the kelch-like ECH-associated protein 1 (Keap1)/Nrf2/antioxidant response element antioxidant pathway. In the present study, the results showed that compared with those in the control group, the protein levels of Nrf2 and HO-1 were significantly reduced in the DCM group, whereas PAR1 protein levels showed no significant alteration; by contrast, the protein levels of PAR1, Nrf2 and HO-1 were significantly increased in the DCME group compared with those in the DCM group.

According to this study, moderate-intensity aerobic exercise has clear guiding significance for the clinical exercise management of patients with DCM. It can inhibit OS by activating the PROC/PAR1/Nrf2/HO-1 pathway, thereby improving DCM. Clinically, the intensity parameters of this exercise are as follows: choose low-to-moderate impact exercises such as brisk walking and jogging, 3-5 times a week, 30–45 min each time, with heart rate maintained at 60%–70% of “220 minus age” (Magutah et al., 2022). This protocol is easy to implement, highly safe, and can help improve DCM. In conclusion, aerobic exercise may alleviate DCM through the aforementioned pathway; the study results lay a foundation for exploring how exercise regulates OS and affects DCM progression, and also provide new perspectives for disease diagnosis and prognosis.

However, the present study had certain limitations. Firstly, the GEO database was used to analyze the key genes involved in aerobic exercise-induced DCM improvement. Notably, the present study used a mixed dataset of rats (GSE4745, GSE6880, GSE5606) and mice (GSE4616) to analyze DEGs. Integrating data from these two species is an effective approach in bioinformatics and systems biology, especially when studying conserved biological mechanisms. This is because rats and mice have notable genetic and physiological similarities, which makes cross-species analysis valuable for improving the robustness and universality of research results. When integrating rat and mouse diabetic cardiomyopathy (DCM) data, the core potential limitation of this study is that the inherent differences in DCM pathophysiological traits between the two species cannot be fully eliminated, which may compromise the accuracy of gene expression analysis. To address this, targeted strategies were adopted during data processing, including only genes significantly differentially expressed (|log_2_FC|>1, P < 0.05) in both species to exclude interference from species-specific genes, prioritizing genes with consistent expression trends, and focusing on core, functionally conserved pathways (e.g., Nrf2/HO-1, PROC/PAR1 pathways) shared by the two species to improve conclusion reliability (Cheng et al., 2024; Sun et al., 2021). Future studies could further optimize the design by using a single species with an increased sample size or adopting advanced bioinformatics techniques to minimize inter-species differences and reduce potential biases (Li et al., 2024; Deng et al., 2024).

## 5 Conclusion

**FIGURE 10:**
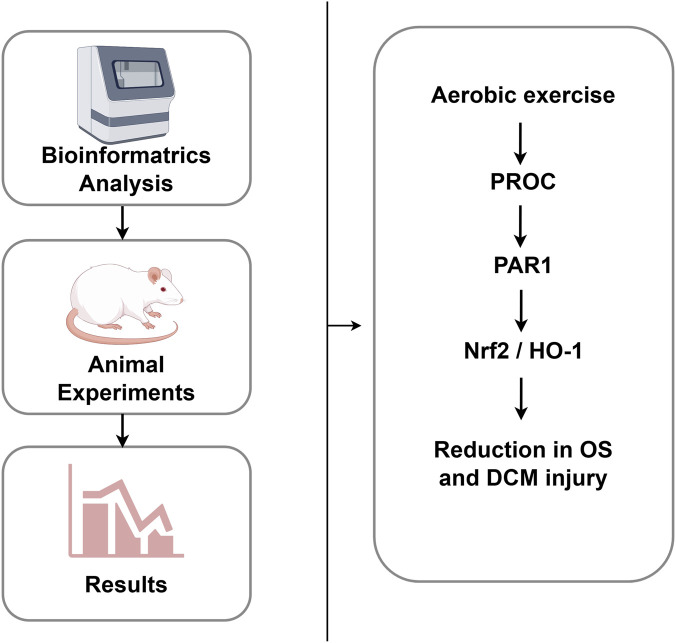
Schematic diagram of aerobic exercise alleviating oxidative stress and diabetic cardiomyopathy (DCM) via the PROC/PAR1/Nrf2/HO-1 signalling pathway. Aerobic exercise alleviates DCM by targeting the hub gene PROC, as identified through bioinformatics and animal models. Mechanistically, aerobic exercise upregulates PROC expression and activates the PROC/PAR1/Nrf2/HO-1 axis, leading to enhanced antioxidant capacity, reduced myocardial injury, and improved cardiac function. PROC, protein C; PAR1, proteinase-activated receptor 1; Nrf2, nuclear factor (erythroid-derived 2)-like 2; HO-1, heme oxygenase-1; DCM, diabetic cardiomyopathy.

Diabetic cardiomyopathy (DCM) is closely linked to oxidative stress (OS). This study combined bioinformatics and animal experiments to confirm that 8 weeks of moderate-intensity aerobic exercise alleviates DCM in rats. Bioinformatics identified nine hub genes, with protein C (PROC) as the key target. Exercise upregulated PROC, activated the PROC/PAR1/Nrf2/HO-1 pathway, improved cardiac function, reduced myocardial injury markers and enhanced antioxidant capacity (Figure 10). Limitations include cross-species data integration. These findings highlight PROC as a novel target and aerobic exercise as a safe DCM intervention, providing insights for clinical management.

## Data Availability

The data generated in the present study may be requested from the corresponding authors.

## Glossary

BNP: brain natriuretic peptide
BP: biological process
c-TnT: cardiac troponin T
CC: cellular component
CK-MB: creatine kinase MB
DCM: diabetic cardiomyopathy
DCME: DCM plus exercise
DEGs: differentially expressed genes
ELISA: enzyme-linked immunosorbent assay
FBG: fasting blood glucose
FINS: fasting plasma insulin
GEO: Gene Expression Omnibus
GO: Gene Ontology
GSH: glutathione
HFD: high-fat diet
HO-1: heme oxygenase-1
HOMA-IR: homeostatic model assessment of insulin resistance
ISI: insulin sensitivity index
KEGG: Kyoto Encyclopedia of Genes and Genomes
LDH: lactate dehydrogenase
logFC: log2 fold change
LVEF: left ventricular ejection fraction
LVFS: left ventricular fractional shortening
LVIDd: left ventricular internal diastolic dimension
LVIDs: left ventricular internal dimension systole
MDA: malondialdehyde
MF: molecular function
Nrf2: nuclear factor (erythroid-derived 2)-like 2
OS: oxidative stress
PCA: principal component analysis
PPI: protein-protein interaction
PROC: protein C
ROS: reactive oxygen species
SOD: superoxide dismutase
STRING: Search Tool for the Retrieval of Interacting Genes
STZ: streptozotocin
